# Yin and Yang: A pair of miRNA modules antagonistically regulate xylem development

**DOI:** 10.1093/plcell/koaf016

**Published:** 2025-01-16

**Authors:** Meenu Singla-Rastogi

**Affiliations:** Assistant Features Editor, The Plant Cell, American Society of Plant Biologists; Department of Biology, Indiana University, Bloomington, IN 47405, USA

Over 400 million years ago, the emergence of vascular plants (tracheophytes) marked a pivotal milestone in the evolution of land plants. The development of specialized vascular tissues, including xylem and phloem, enabled the plants to thrive terrestrially. Xylem, composed primarily of tracheary elements (TEs), is responsible for transporting water and minerals from the roots to the rest of the plant body. Notably, TEs are functionally dead cells, optimized for efficient water conduction. The formation of xylem TEs involves a multi-step differentiation of meristematic cells. This intricate developmental process is tightly regulated by a network of hormones, transcription factors, and degrading enzymes (Heo et al. 2017). Disruptions in this complex regulatory system can lead to severe defects in xylem development, thereby impacting overall plant growth and development.

To achieve mature and fully functional xylem TEs, plants meticulously regulate gene expression during various stages of meristematic cell differentiation. Key players in this process are microRNAs (miRNAs), small RNA molecules (20–24 nucleotides long) that control gene expression by binding to complementary sequences on target mRNAs. This binding can lead to mRNA degradation (cleavage) or prevent protein translation (suppression), effectively silencing the target gene. While many miRNAs have been shown to influence plant growth and development, including xylem development, most studies focus on mature xylem cells (Curaba et al. 2014). Therefore, the changes in the levels of several miRNAs contributing to a streamlined transition at each stage of xylem development remain unclear (Zhao et al. 2022). In a new work, **Chunhao Liu and colleagues (Liu et al. 2024)** elucidated the dynamic role of 2 antagonistically operating miRNA modules during the complex process of TE differentiation.

Looks can be deceiving, but not in this case. To gain insights into the role of different miRNAs in TE development, the researchers investigated changes in miRNA accumulation profiles (the “miRNome”) during different stages of TE differentiation with the help of a dynamic visualization tool Vascular cell Induction culture System Using Arabidopsis Leaves (VISUAL). This analysis facilitated the identification of distinct stages of TE differentiation and the precise collection of RNA samples for small RNA sequencing and further analysis. Through establishing a comprehensive miRNA coexpression regulatory network, the authors identified 2 distinct miRNA modules—a positive regulatory module (module II) and a negative regulatory module (module I)—hypothesizing that these 2 miRNA modules work in opposition to regulate the complex process of xylem development. To further investigate this hypothesis, the authors functionally analyzed TE differentiation in the knockout mutant and overexpression lines of 2 selected candidate miRNAs, miR408 and miR163, belonging to modules II and I, respectively (Figure A). The results indicated that miR408 and miR163 exerted a positive and negative impact on TE differentiation (Figure B and C) by promoting cambium formation and inhibiting secondary cell wall formation, respectively (Liu et al. 2024). These results corroborated the findings of previous studies, validating the reliability and importance of studying the miRNA coexpression network in TE differentiation.

**Figure.**
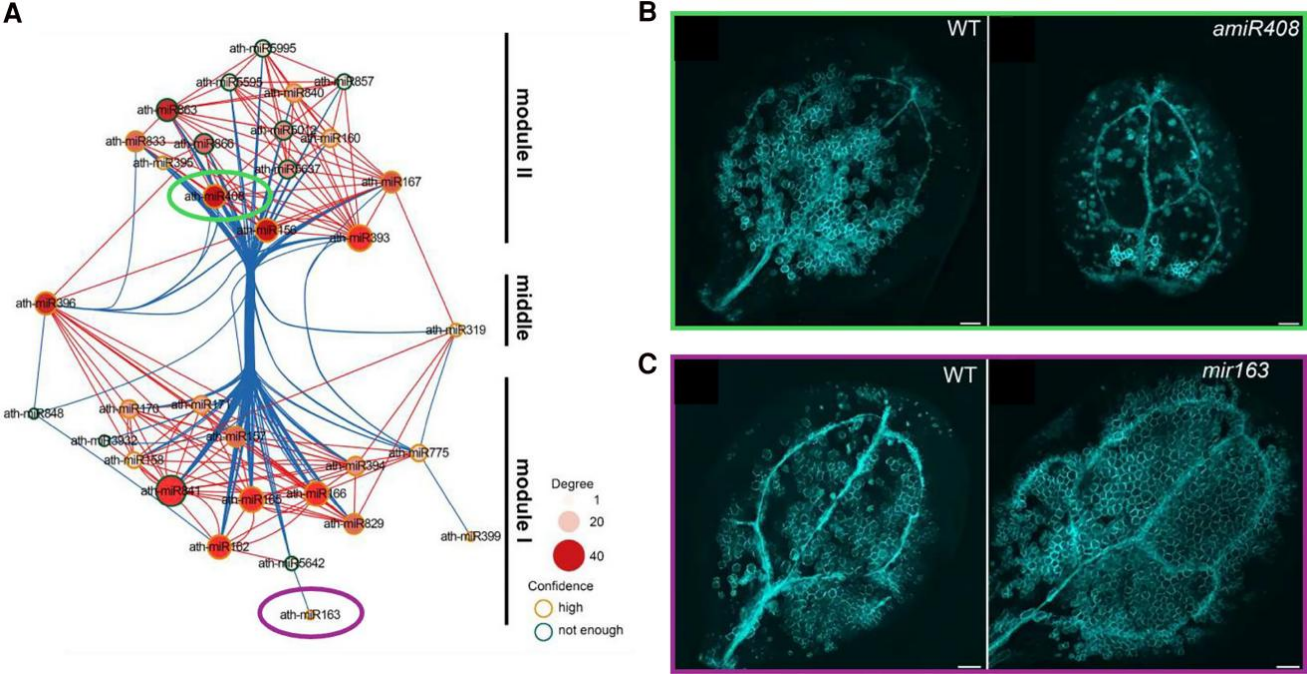
Role of distinct miRNA modules in xylem TE development. **A)** miRNA coexpression network during the process of TE differentiation. **B, C)** The roles of 2 contrasting miRNAs, miR408 (module II) and miR163 (module I), in TE differentiation and vascular tissue development were assessed in WT, *amiR408* (a transgenic line overexpressing an artificial microRNA that targets the pre-miR408 sequence), and *mir163* plants at 72 h after induction (hai) in VISUAL, respectively. Scale bar, 200 *μ*m. Adapted from Liu et al. (2024), Figures 4D, 5 A and B, and 6 A and B.

The authors extended their investigation to poplar (*Populus tomentosa*) and found that xylem development is also regulated by 2 contrasting miRNA modules in this distantly related woody plant species, similar to what was observed in Arabidopsis (Liu et al. 2024). These findings support the idea that this contrasting miRNA-mediated regulatory mechanism is conserved across plant species. The findings of this study provide novel insights into the evolutionarily conserved role of antagonistically operating regulatory small RNAs in plant development.

To broaden the relevance of distinct sets of contrasting miRNA regulatory modules in other plant developmental processes, the authors curated publicly available Arabidopsis miRNome data from the studies on pollen and embryonic development. Through miRNA coexpression network analysis, they confirmed the existence of 2 highly negatively correlated miRNA modules during pollen development, whereas only 1 positive coexpression module during embryonic development was identified. This observation leads to the broader question of the selective role of antagonistic miRNA modules in different processes of plant development. Most researchers focus on a unique developmental stage to build a coexpression network; however, this study by Liu et al. (2024) provides a benchmark for future studies aiming to elucidate miRNA function in plant development.

## Data Availability

No new data were generated or analyzed in support of this research.

## Dive Curated Terms

The following phenotypic, genotypic, and functional terms are of significance to the work described in this paper:

mir163 Gramene: AT1G66725
mir163 Araport: AT1G66725

xylem AmiGo: PO:0005352

## References

[koaf016-B1] Curaba J, Singh MB, Bhalla PL. miRNAs in the crosstalk between phytohormone signalling pathways. J Exp Bot. 2014:65(6):1425–1438. 10.1093/jxb/eru002 24523503

[koaf016-B2] Heo JO, Blob B, Helariutta Y. Differentiation of conductive cells: a matter of life and death. Curr Opin Plant Biol. 2017:35:23–29. 10.1016/j.pbi.2016.10.007 27794261

[koaf016-B3] Liu C, Li A, Guo Z, Chen N, Wang Y, Tang W, Wu Y, Liu J, Wang Z, Li L, et al miRNome reveals two antagonistic microRNA modules with different functions in regulating xylem tracheary element development. Plant Cell. 2024. 10.1093/plcell/koaf011 PMC11760538 39792476

[koaf016-B4] Zhao W, Meng X, Xu J, Liu Z, Hu Y, Li B, Chen J, Cao B. Integrated mRNA and small RNA sequencing reveals microRNAs associated with xylem development in *Dalbergia odorifera*. Front Genet. 2022:13:883422. 10.3389/fgene.2022.883422 35547261 PMC9081728

